# Comparison of design methods for negative pressure gradient rotary bodies: A CFD study

**DOI:** 10.1371/journal.pone.0228186

**Published:** 2020-01-30

**Authors:** Pingan Liu, Hancong Liu, Yanxi Yang, Mengjun Wang, Yangguang Sun

**Affiliations:** 1 College of Aerospace and Civil Engineering, Harbin Engineering University, Harbin, China; 2 Key Laboratory of dual dielectric Power Technology, Heibei Hanguang Industry Co. Ltd. Handan, China; Tongji University, CHINA

## Abstract

Computational fluid dynamics (CFD) simulation is used to test two body design methods which use negative pressure gradient to suppress laminar flow separation and drag reduction. The steady-state model of the Transition SST model is used to calculate the pressure distribution, wall shear stress, and drag coefficient under zero angle of attack at different velocities. Four bodies designed by two different methods are considered. Our results show the first method is superior to the body of Hansen in drag reduction and the body designed by the first method is more likely to obtain the characteristics of suppressing or eliminating separation, which can effectively improve laminar flow coverage to achieve drag reduction under higher Reynolds number conditions. The results show that the negative pressure gradient method can suppress separation and drag reduction better than the second method. This successful design method is expected to open a promising prospect for its application in the design of small drag, small noise subsonic hydrodynamic hull and underwater weapons.

## Introduction

Energy conservation and emission reduction is an eternal topic in the development of vehicle design optimization technology. People usually adopt active control to improve energy efficiency for achieving energy saving and emission reduction. By using special shape, drag can be reduced and more energy will be saved in turn.

For laminar unseparated shapes, they can effectively reduce the drag and vehicle noise, and increase the wing lift. Many studies have reported the feasibility of using active flow control methods to avoid separation and delay laminar flow to turbulent transitions [1–5]. These active control methods are typically based on suction/blowing, drag-reduction solution, surface cooling/heating, different shape transformations, or even electromagnetic forces. Proper use of these methods can delay the transition from laminar to turbulent flow patterns, inhibiting or even eliminating flow separation. However, as far as we know all above methods require extra energy to work.

Passive flow control method which is based on the shape and surface flow characteristics of the rigid body only. The shape and flow characteristics are tuned to control the surface flow state. Although not as effective and accurate as the former methods, the advantage is that no external energy is consumed.

Inspired by the shape of dolphins (during inertial motion without maneuver and shape change) [6], Nesteruk et al. developed a typical streamlined shape UA2 [7] (similar to animal shape [8]). Their results indicate that drag can be reduced only when body shape and flow pattern match.Their work inspire us that the biological shape is one of the best choices to meet this criterion.

The Institute of Fluid Mechanics (IHM) of the National Academy of Sciences in Kiev, Ukraine, studied the possibility of achieving non-separating flow on rigid bodies. The surface flow of streamlined body UA-2 was experimentally studied at the University of Technology, Brunswick (Tub) [7]. These experiments proved using non-separated shapes could improve vehicle performance effectively. Specially shaped bodies optimized for reducing drag calculated and tested in [9–15]. Ref.[16] provided a detailed description of the theoretical and experimental results of the attachment flow on the surface of a rigid body. Ref.[17] tested the performance of special shapes with different aspect ratios by theoretical method, and proposed a theoretical estimation method of laminar maximum velocity. However, the validity of the method was in doubt, and further numerical and experimental methods needed to demonstrate it.

Morteza Anbarsooz proposed a new method to reduce the drag of underwater hulls that the fluid slip phenomenon of the Anbarsooz 7 hydrophobic surface could significantly reduce the surface frictional drag on the surface of non-separators [18]. Ling et al. found that the Reynolds stress at the top of the roughness is much lower than the viscous stress when the roughness effect was not dominant. The theoretical relationship between drag reduction and sliding length in turbulent boundary layer was determined [19]. Ceccio SL et al. studied the drag reduction effect of ventilated supercavitation on underwater vehicle [20]. Shereena et al. studied drag reduction of axisymmetric underwater body by computational fluid dynamics (CFD). Their results showed that the best drag reduction could be obtained when the influence parameters were in a certain range[21]. Thomas C. Corke et al summarized the flow control methods for reducing turbulence in recent decades[22]. Karsten Oehlert et al studied the prediction capabilities of three RANS turbulence models based on the flow of NREL S809 wing at various angles of attack. The results showed that both the kkL-ω and γ-ReΘ models show sufficiently accurate predictions of transition start positions[23]. Yiqing Li et al studied a new a new local gradient active control optimization method based on simplex downhill algorithm which greatly improved computing efficiency and optimization effect by RANS solver[24]. Yuze Jiang et al studied the main characteristics of drag reduction by grooved surface through RANS solver and verify the effectiveness of the numerical model and the drag reduction performance of the trench section through the experimental results[25]. Grzegorz Filip et al studied the influence of the vortex generators by using RANS, LES and DES tubulence models and compared with the physical measurement results[26]. S. M. A. Aftab et al investigated the effect of sine and spherical tubercle leading edges on wing surface flow through Transition SST tubulence model. The CFD and experimental results showed that the spherical TLE could effectively improve the performance of the wing[27]. Katherine Yates et al studied an active surface pulsed DC plasma actuator array which can effectively reduce the viscous resistance in the turbulent boundary layer with zero pressure gradient under adverse pressure gradient conditions[28].

In this paper, we evaluate two negative pressure gradient control separation design methods by CFD. The advantanges and disadvantages of both methods are evaluated by comparing the elastic surface flow pattern designed by the two methods, and the magnitude and variation of the drag coefficient.

## Research method

### Main characters of the special bodies of revolution

The negative pressure gradient special body of revolution can be calculated by two methods [16]. The first method describes continuous surface was represented as follows:

$$R\left(x\right)=\left\{ \begin{array}{l} sqrt\left(Ex^{2}\left(ax+6c\right)\right),0\leq x<x_{*}\\ sqrt\left(Ea_{1}{\left(x-1\right)}^{3}\right),x_{*}\leq x\leq 1, \end{array}\right.$$ (1)

$$c=-\frac{a_{1}{\left(x_{*}-1\right)}^{2}}{2x_{*}^{2}},a=\frac{a_{1}{\left(x_{*}-1\right)}^{2}\left(x_{*}+2\right)}{x_{*}^{3}},\;E=\frac{1}{6\;\ln\varepsilon }$$ (2)

$$C_{p}=-\ln\varepsilon \frac{d^{2}R^{2}\left(x\right)}{dx^{2}}$$ (3)

Different closed (*R(L) = 0*) and unclosed (*R(L)>0*) shapes (*L* is body length) can be obtained by changing the values of constant parameters x_*_, *a*, *a*_*1*_, *c*.

The second method describes continuous pressure distribution had to use source and sink correction because of surface discontinues. The stream function of the axisymmetric potential flow of the inviscid incompressible fluid was represented as follows

$$\begin{array}{l} \varphi \left(x,r\right)=0.5r^{2}+\beta_{1}u\left(x_{*}\right)-0.75E\left\{a\left[F_{1}\left(x_{*}\right)-F_{1}\left(0\right)\right]\right\}+a_{1}\left[F_{1}\left(1\right)-F_{1}\left(x_{*}\right)\right]+\\ +2\left(ax+2c\right)\left[F_{2}\left(x_{*}\right)-F_{2}\left(0\right)\right]+2a_{1}\left(x-1\right)\left[F_{2}\left(1\right)-F_{2}\left(x_{*}\right)\right] \end{array}$$ (4)

$$\beta_{1}=0.75E\left[ax_{*}^{2}+4cx_{*}-a_{1}{\left(x_{*}-1\right)}^{2}\right]$$

$$u\left(s\right)=\sqrt{r^{2}+{\left(s-x\right)}^{2}},\;F_{1}\left(s\right)=2u^{3}\left(s\right)/3$$

$$F_{2}\left(s\right)=0.5\left(s-x\right)u\left(s\right)+0.5r^{2}\ln\left[s-x+u\left(s\right)\right]$$

$$c=\frac{a_{1}\left(x_{*}-1\right)\left(1-2x_{*}\right)}{4x_{*}^{2}},\;a=\frac{a_{1}\left(x_{*}-1\right)\left(2x_{*}^{2}+2x_{*}-1\right)}{2x_{*}^{3}},\;E=\frac{1}{6\ln\varepsilon }$$ (5)

Where *x*, *r* are cylindrical coordinates. The corresponding axisymmetric body radius *R(x)*, flow components *v*_*x*_, *v*_*r*_ and pressure coefficient on the surface were calculated with the use of following equations:

$$\psi \left(x,R\left(x\right)\right)=0,\nu_{x}=\frac{1}{r}\frac{\partial \psi }{\partial x},\nu_{r}=-\frac{1}{r}\frac{\partial \psi }{\partial r}$$ (6)

$$C_{p}\left(x\right)=\frac{2\left(P-P_{\infty }\right)}{\rho U_{\infty }}=1-\nu_{x}^{2}\left(x,R\left(x\right)\right)-\nu_{r}^{2}\left(x,R\left(x\right)\right)$$ (7)

Where *p* is the pressure of body surface, and *p*_*∞*_ is the infinite pressure, *ρ* is the density of the fluid and *U*_*∞*_ is the ambient flow velocity of the fluid. Varying the values of constant parameters x_*_, *a*, *a*_*1*_, *c* different closed (*R(L) = 0*) and unclosed (*R(L)>0*) shapes can be obtained (*L* is the body length). In the latter part, we note it as the second method.

In order to predict the flow separation phenomena in real flow by theoretical pressure distribution, the following critical equations are proposed for the point of separation on the body of revolution [29]:

$$\frac{16.29}{R^{2}U_{e}^{5.045}}\frac{dU_{e}}{dx}\int_{0}^{x}R^{2}\left(\xi \right)U_{e}^{4.045}\left(\xi \right)d\xi +5.4575=0$$ (8)

Where *U*_*e*_*(x)* is the velocity of the inviscid flow at the outer edge of the boundary layer. Eq (5) and theoretical pressure distribution from exact solution Eq (2) and Eq (3) can be used to solve the point of separation in incompressible fluid.[16]

Nesturuk’s works in unseparated body may be a good measure to solve the energy conservation of vehicle. However, he abandoned the first method because he thought it is impossible to occur pressure discontinue in subsonic flow and put his focus on the second method which has continues pressure distribution.

**Fig 1:**
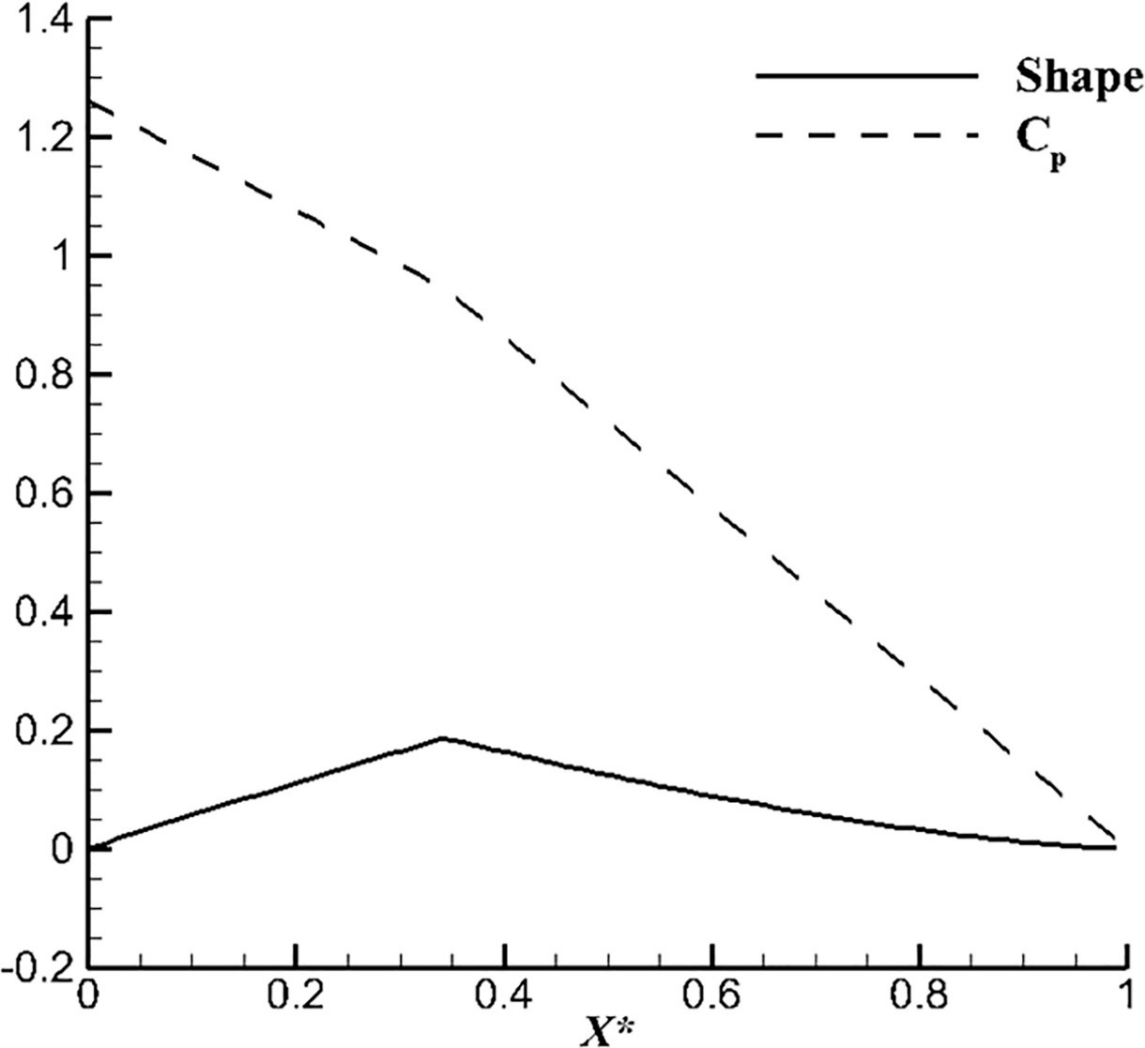
Initial surface and *C*_*p*_ design by the second method.

Fig 1 shows uncorrected surface and *C*_*p*_ calculated by the second method. In one sense, we think it is a pity to abandon the first method because the error between Eq (3) and exact solution may be responsible for leading pressure discontinue. The pressure discontinue is caused by mathematical errors but not a real physical phenomenon. In other words, we think the first method also has its own potential to become a better solution.

**Fig 2:**
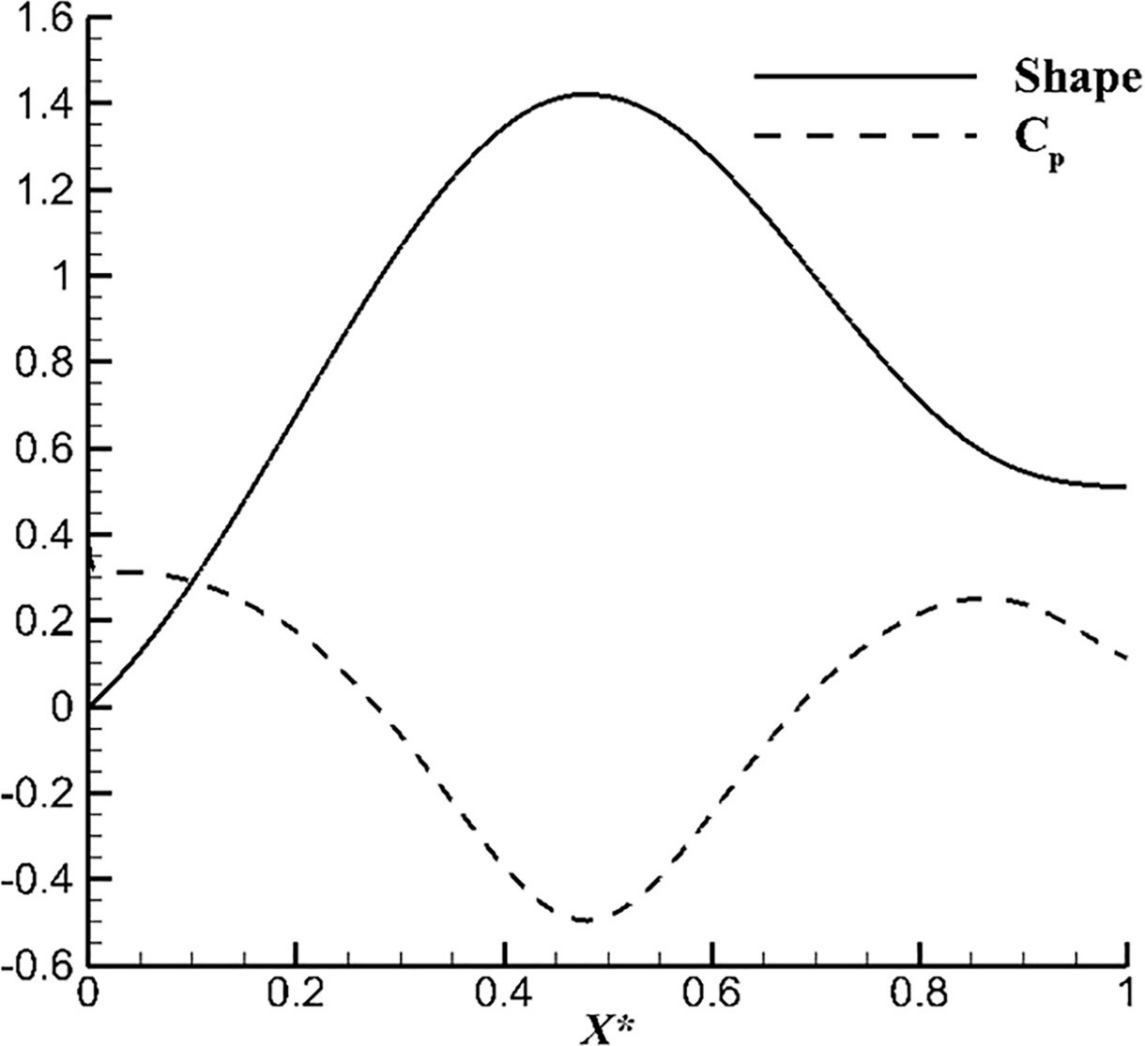
*C*_*p*_ and shape of the modified surface.

According to the equation characteristics, the starting point of the second method is the full negative gradient. However, to satisfy this condition, the value of x_*_ which determines the maximum diameter position must be limited. For example, the x_*_ should be less than 0.366 when *R(L) = 0*. If the maximum diameter is too close to the head of the body, the separation possibility will be greatly improved. Velocity attenuation occurs when an incoming stream strikes the nose. There will produce two different results when the maximum diameter point is too forward. One is that velocity recovery time becomes shorter because velocity recovery zone is generated by negative pressure gradient before the maximum diameter point. As well known, flow separation zone is caused by positive pressure gradient and fluid viscosity. The other one is excessive length of positive gradient region is liable to cause flow separation. The positive pressure gradient at the back of the maximum diameter position leads velocity loss and flow separated eventually, because the position of the maximum diameter point is limited and the ability of the second method to handle separation will also be limited. Fig 2 shows the modified surface and *C*_*p*_. It can be seen that there is still a positive pressure gradient on the surface of the optimized body.

There are two advantages for the first method. One advantage is the maximum diameter point is not limited while maintaining the assumption of full negative pressure gradient. From the aspect of the surface characteristics, the original intention of the design is to maintain a negative pressure gradient. Therefore, there will be no separation before the point of the maximum diameter. In order to effectively extend the negative pressure gradient zone, delay the position of the maximum diameter point would be a good choice. At the same time, velocity recovery from the negative pressure gradient zone before the maximum radius point can effectively avoid the separation. Subsequently, separation suppression and evasion are more directed to the laminar boundary layer and flow separation is more difficult in turbulent boundary layer than in laminar boundary layer. As a result, the maximum diameter point could not infinitely delayed, and the turbulent transition phenomenon caused by the excessive length of the acceleration section should be guarded against. Another advantage is the body surface will be continuous without modifications which reduce the damage to the real physical solution caused by the correction process. From the modified result of the second method, the initial design is a continuous negative pressure gradient (because the error between the Eq (3) and the exact solution of *C*_*p*_ may not be caused by a real physical phenomenon), the final corrected *C*_*p*_ has a positive pressure gradient but not a continuous negative pressure gradient. From the point of physical essence, there must be a positive gradient on a continuous surface (surface stops at the maximum radius except). Therefore, if we aim to obtain a streamlined continuous surface, the first method seems to be an effective and reasonable choice.

From a theoretical view, the first method has good application prospects and is computationally efficient. The starting point of the second method is more excellent. However, the initial setting was broken during the solution of the surface correction. The final design which has positive pressure gradient and loss of computational efficiency and limited design is the same as the first one.

**Fig 3:**
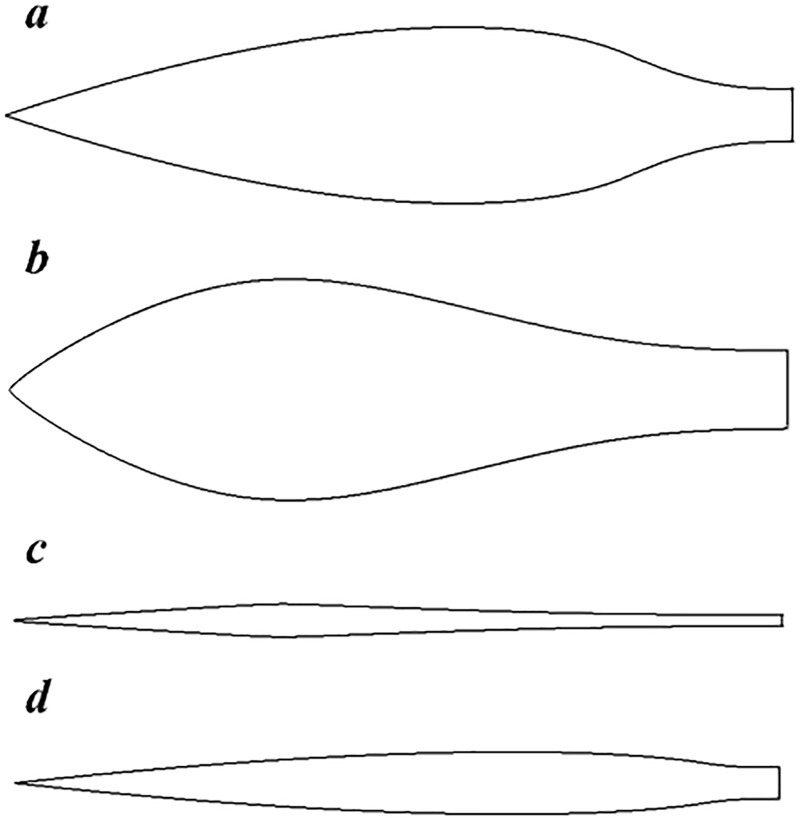
Body shapes designed by different methods. From (a) to (d): the body of U-4, UA2, U100 and U-13.

Fig 3 shows the bodies designed by these two methods. U-4 which is designed by the first method is similar to the body of Hansen [14] [16]. The length, tail diameter and maximum diameter of U-4 and position of the maximum diameter the that are the same as those of Hansen. U-13 is also designed by the first method. The length and maximum diameter of it is 1.5 m and 0.122 m respectively. The tail of U-13 is half of the maximum diameter of the body. U-100 and UA2 are designed by the second method [7] [17], the L/D of U-100 is 23.3 and which for the UA2 is 3.6, and tail of body is one-three of the maximum diameter of the body. U-100 is as long as U-13. L is the length of body and D denotes the maximum diameter of the body.

### Computational fluid dynamics method description

**Fig 4:**
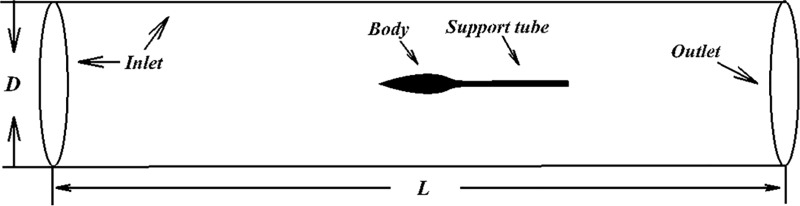
Computational domain of body.

To reflect the physical process in an efficient and intuitive way, we decide to use computational fluid dynamics (CFD) research practical value of the two methods. In order to efficiently and realistically simulate the surface flow, Transition SST (Shear Stress Transfer) turbulence model is adopted to steady simulate different bodies. The computational domain is shown in Fig 4, D is 10 times of the maximum diameter and L is 6 times of the body length. Velocity-inlet boundary condition and pressure-outlet boundary condition are used in inlet and outlet respectively. The body and support tube are non-slip wall. The length of support tube is as long as the body. In order to calculate incompressible flow efficiently, the pressure-based separated algorithm is used. The third-order QUICK scheme is employed to discretize the convective and diffusive terms, and we use Semi-Implict Method for Pressure Linked Equation Consistent (SIMPLEC) algorithm is used for the velocity-pressure decoupling. The water density is set as 1,000 kg/m^3^ and the dynamic viscosity of water is set as 0.001003kg/(m·s).

**Fig 5:**
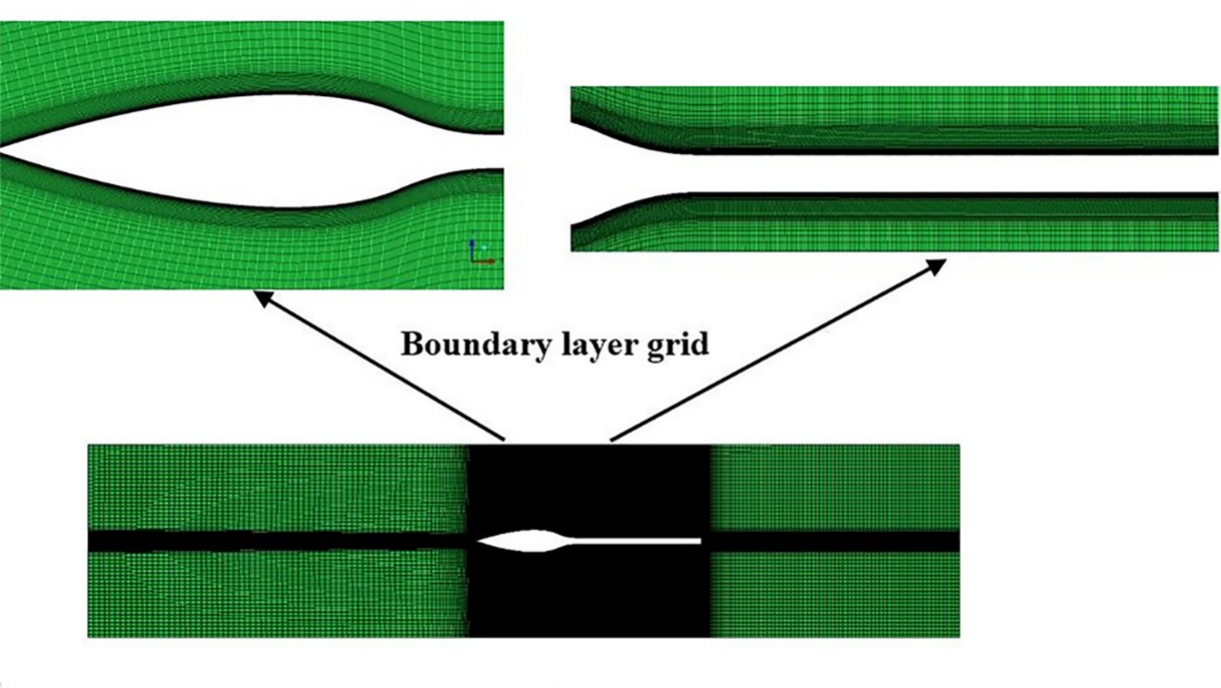
Grid for the body of U-4.

To ensure the calculation accuracy, the Y^+^ of the first layer grids of the boundary layer on the body surface is below 1 and 15–20 layers in the boundary layer are densified (The values of Y^+^ are dependent on the resolution of the mesh and the Reynolds number of the flow, and are defined only in wall-adjacent cells). The mesh independence is also studied, in which the mesh size decreases gradually until no significant change in the total drag coefficient and residual are observed. The number of grid is about 10 million, slightly different with the change of test body. Among them, the grid number of U-4 is about 11 million, the grid number of U-100 is about 8 million, the grid number of UA2 is about 7 million, and the grid number of U-13 is about 6 million. Fig 5 shows the grid details of the body of U-4. Fig 5 shows the grid details of the body of U-4.

**Fig 6:**
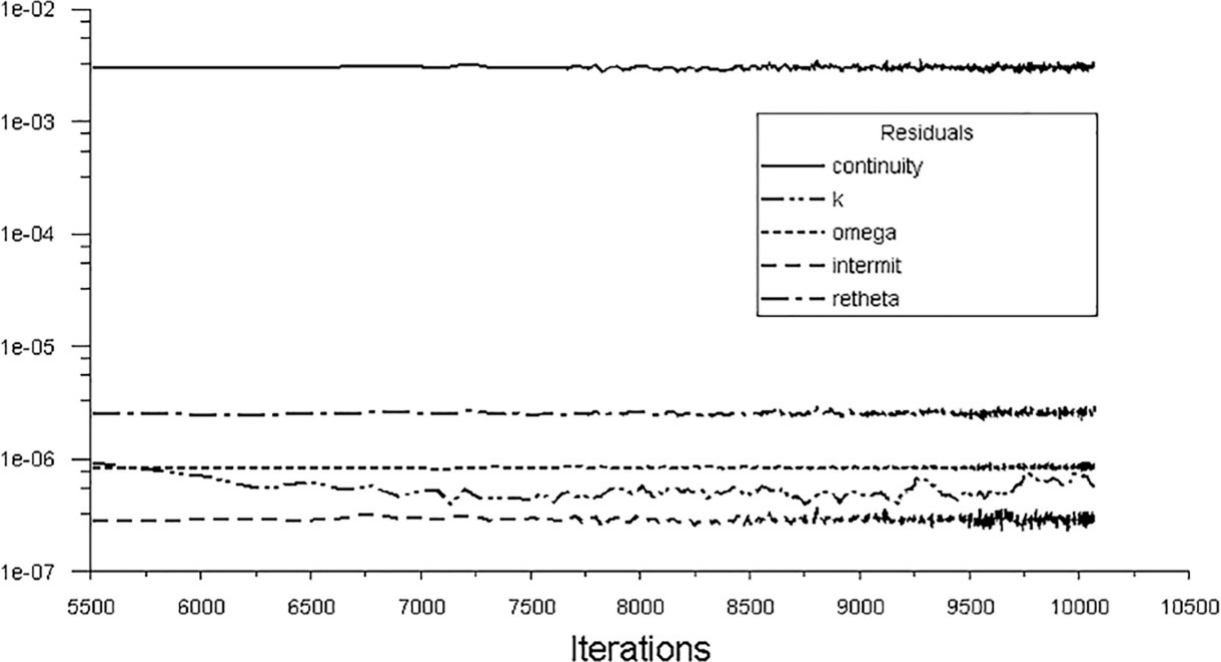
Convergence and divergence criterion.

Fig 6 shows the residual convergence of the transition equation which is used as the convergence criterion for the calculation of the transition model. The residuals of the intermittent factor and Re theta variable converge to less than 1e^-05^ magnitude. Therefore, the transition equation is convergent under such situation. The computational effort required for the simulation is approximately 2400 core hours, which is the product of the number of computational cores and wall-hours.

## Results and discussion

### CFD results for U-4

**Fig 7:**
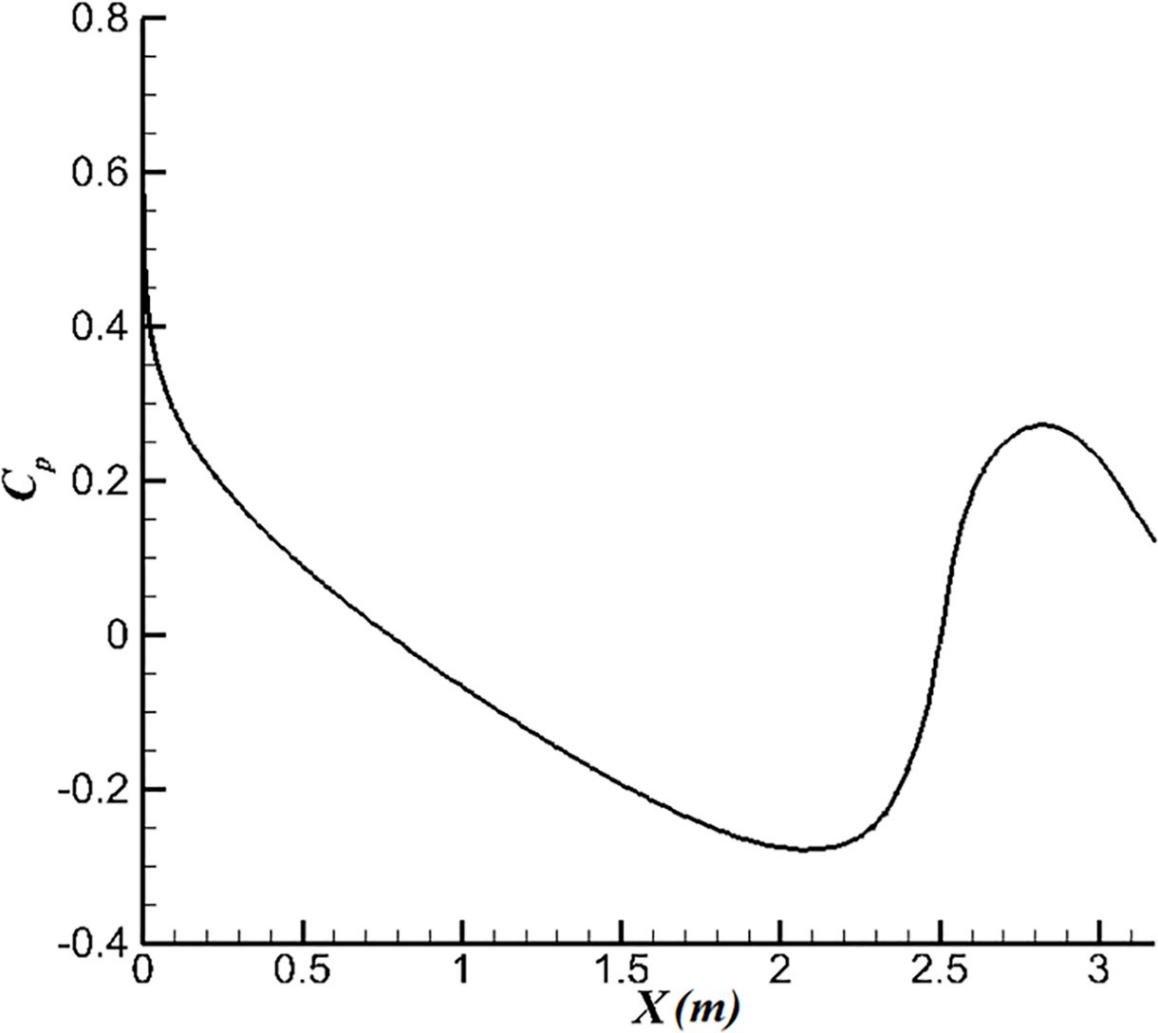
Pressure distributions of the body.

Firstly, we simulate the water flow at different velocities at attack angle *α* = 0° and compare the results with the experimental results for the body of Hansen [14]. Fig 7 shows the full 3D pressure distributions *C*_*p*_ of the body. The pressure distribution of surface tends to be uniform. For the body of U-4 designed by the first method, there is a wide range of negative pressure gradients on its surface and large negative pressure gradient can be obtained again after the fluid passes through a short positive pressure gradient to suppress the separation phenomenon.

**Fig 8:**
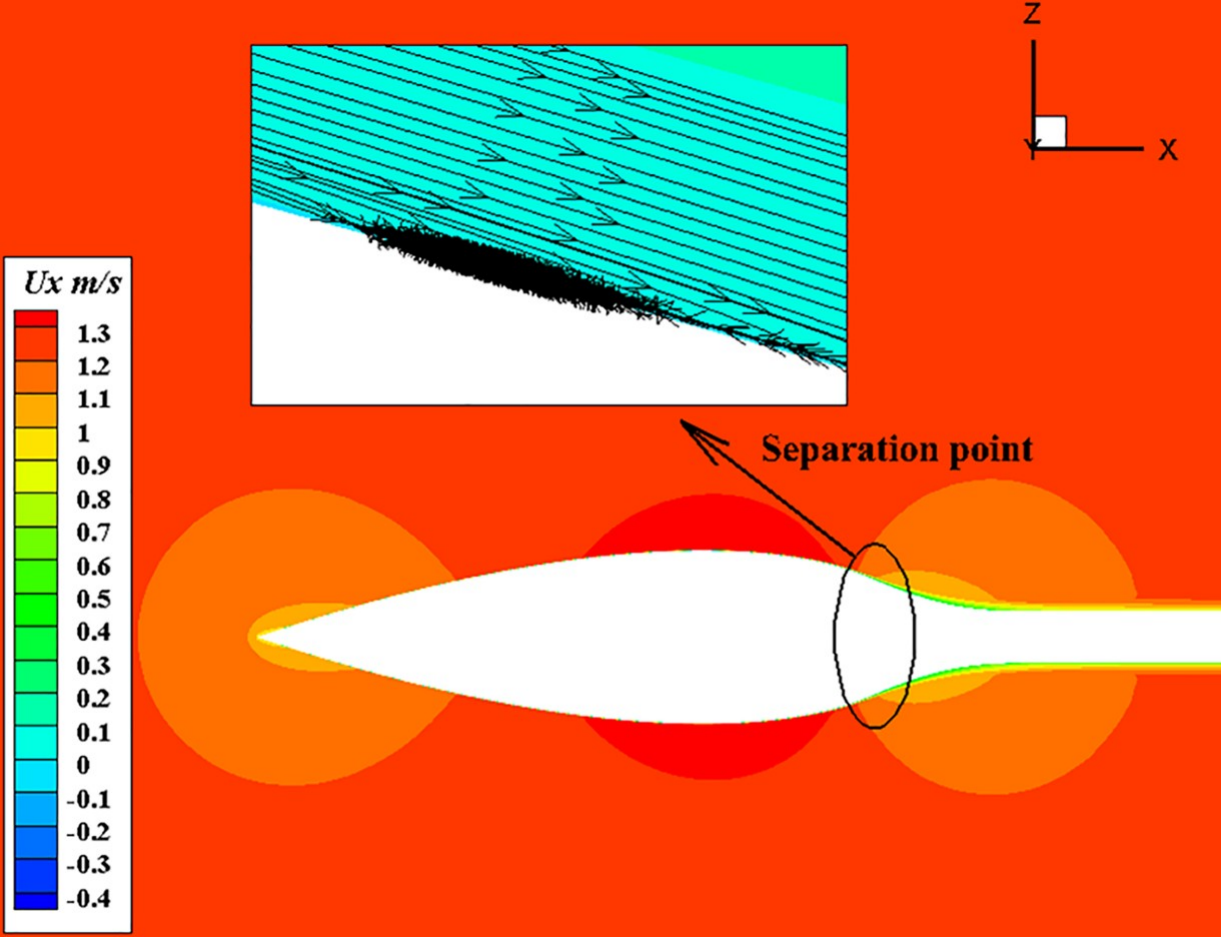
Contours of the axial velocity at U_∞_ = 1.22m/s.

Fig 8 shows the contours of ambient velocity at 1.22m/s for U-4. Velocity attenuation and pressure surge occurs when incoming flow strikes the surface of body. As a result, a stable laminar flow appears on the surface. Fluid flows through the front section of the body, the velocity is gradually picking up as surface pressure drop. After the fluid passing the maximum diameter point, the pressure gradually increases and the velocity decreases in turn. After passing the positive gradient zone, the pressure gradually decreases and the kinetic energy of the fluid gradually recovers. From Fig 8, we found a small separation point in the positive pressure gradient region when the velocity is 1.22m/s. It means that the velocity is too low to overcome the effects of positive pressure gradients and viscous forces.

**Fig 9:**
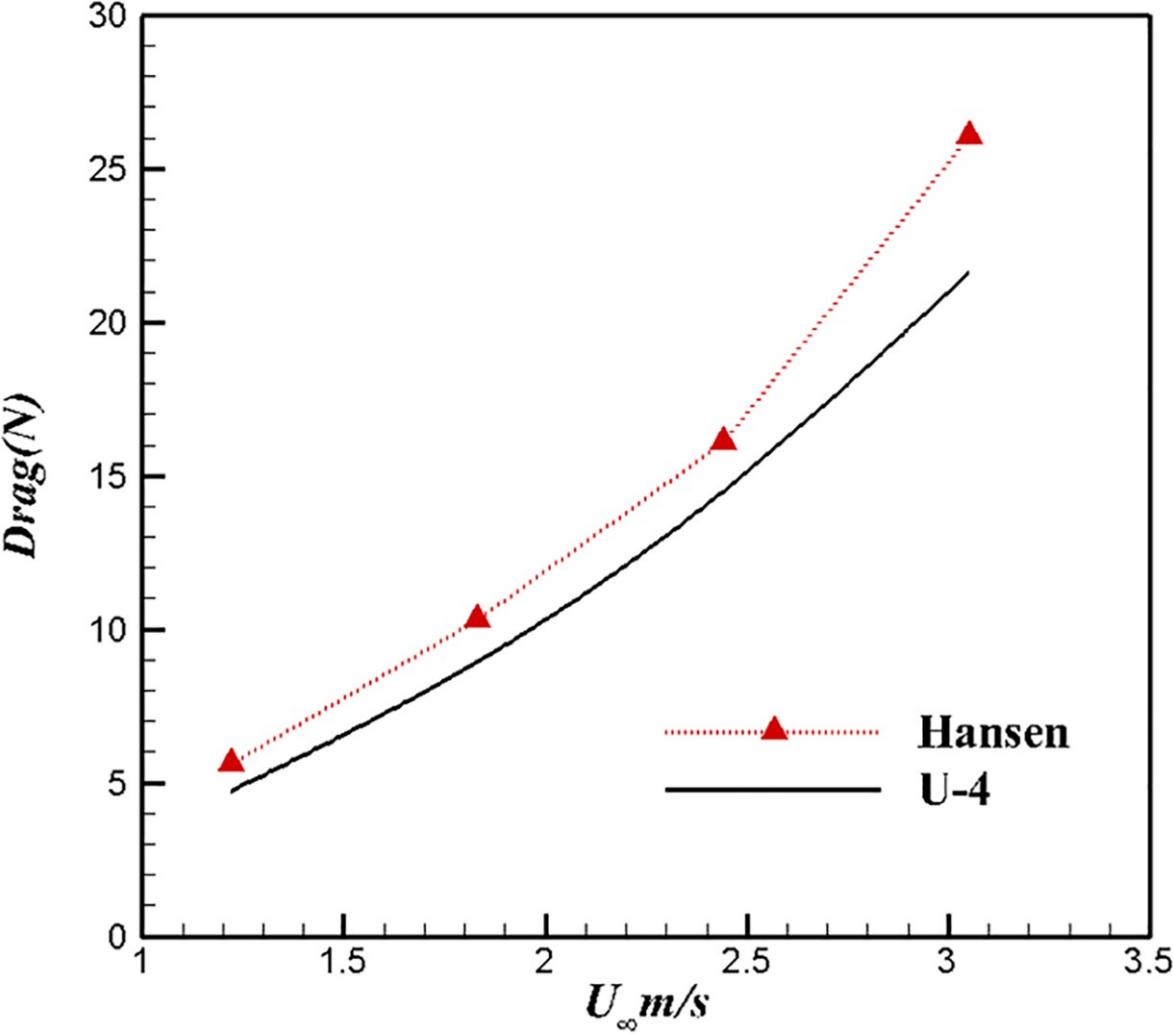
Comparisons of resistance between U-4 and Hansen.

Fig 9 shows the comparison of drag between U-4 and the body of Hansen. The relationship between the drag and inflow velocity of U-4 and Hansen is consistent. The drag of U-4 is less than that of the body of Hansen under the same velocity. The difference in drag between the body of Hansen and U-4 ranges from 10% to 20%.

**Fig 10:**
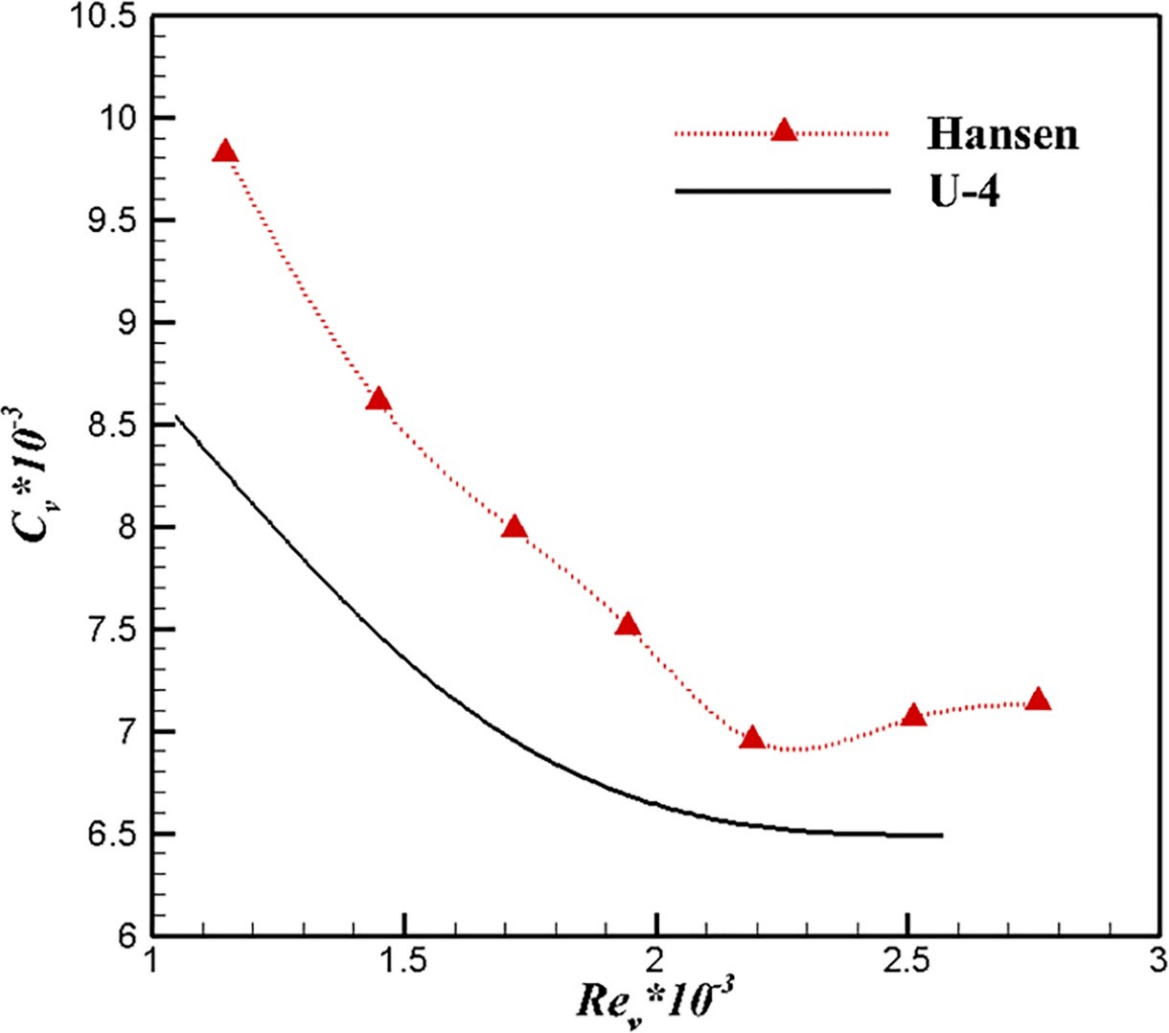
Comparisons of volumetric drag coefficients.

The comparison of volumetric drag coefficients are shown in Fig 10. U-4 shares a similar changing trend with the body of Hansen in the variation of volume drag coefficent. The volume drag coefficient of Hansen rises earlier. Two possible reasons may explain this: The transition point appears in the front of the maximum diameter point, and the drag increases rapidly due to high-speed turbulence. Additionally, the separation on the surface of the body causes an increase in drag. The early transition point of laminar flow caused by the velocity increase leads to a rapid increase of drag. Such phenomenon indicates that the optimal working velocity of U-4 could be higher than that of Hansen. Therefore, U-4 would obtain better drag reduction effect than the body of Hansen.

Volume drag coefficient and volume Reynolds number can be obtained as follows:

CV=2FρU2V23 (9)

Rev=V13Uν (10)

Where F is the total drag of the body. U is the ambient velocity. V is the volume of the body, and *ρ* and *v* are the dencity and kinematic viscosity of the water respectively.

**Fig 11:**
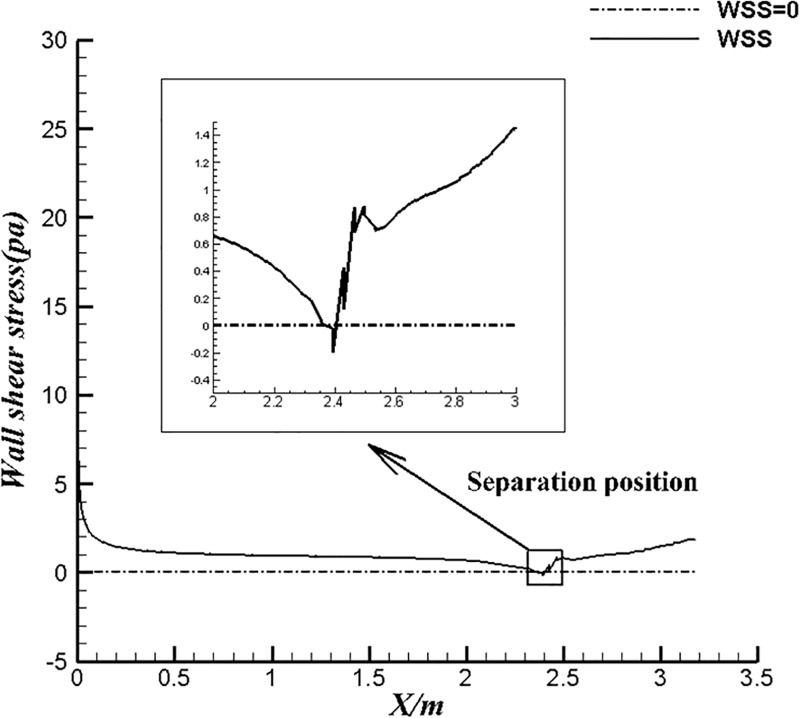
X-Wall shear stress of U-4 at U_∞_ = 1.22m/s in water.

The laminar flow is prone to separate when the velocity is low. Fig 11 shows x-wall shear stress of U-4 at the ambient velocity of 1.22 m/s in water. At this test velocity we found there is some slight separation at the positive pressure gradient region of body. Where x wall shear stress is less than zero indicates that there is separation appeared on surface of the body. Obviously, the kinetic energy of the low-velocity fluid is not sufficient to provide the positive gradient and the consumption of the fluid viscosity eventually leads separations appear.

**Fig 12:**
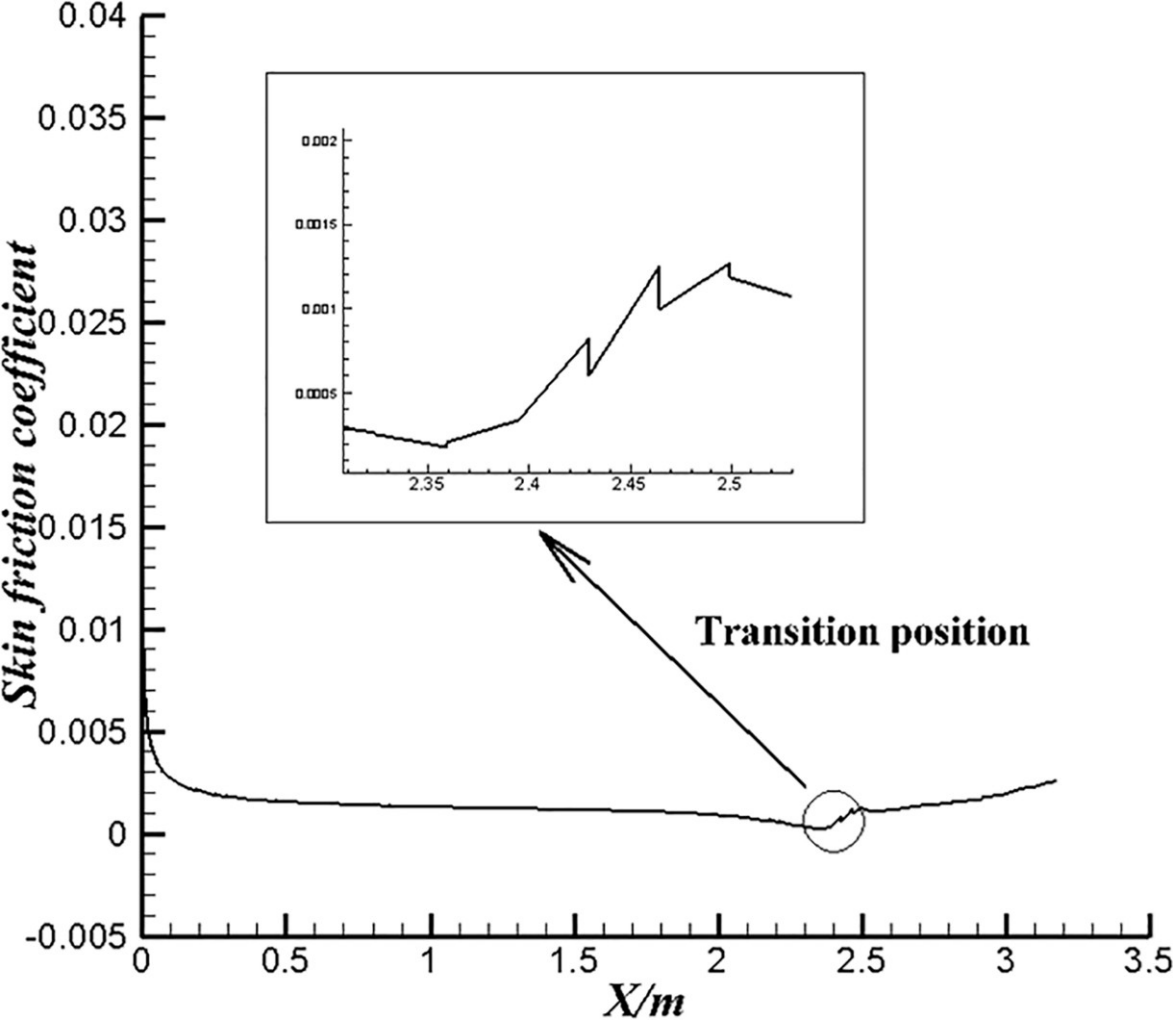
Skin friction coefficient of U-4 at U_∞_ = 1.22m/s.

Fig 12 shows the skin friction coefficient of U-4 at the ambient velocity of 1.22m/s in water. The changing trend is consistent with that of the wall shear stress. As well known, transition occurs at the place where the surface friction coefficient begins to increase. Therefore, it can be considered that instability (e.g., pressure) inside the fluid caused by flow separation and reattachment triggers laminar-turbulent transition. For low-speed incompressible flows, we ignore the influence of temperature effects here. As we all know, the appearance of separation will greatly increase the differential pressure drag. The flow instability and laminar-turbulent transitions caused by flow separation also increase the frictional drag which makes the control of separation points important.

Then we compare the results of U-4, UA2 [7] and U-100. Experiment information is listed in the Table 1.

**Table 1:** Computational Conditions for Comparing body.

Type	Length (m)	Maximum Diameter (m)	Water depths (m)
U-4	3.18	0.71m	2.5m
UA2	3.00	0.88m	2.5m
U-100	3.18	0.14m	2.5m

**Fig 13:**
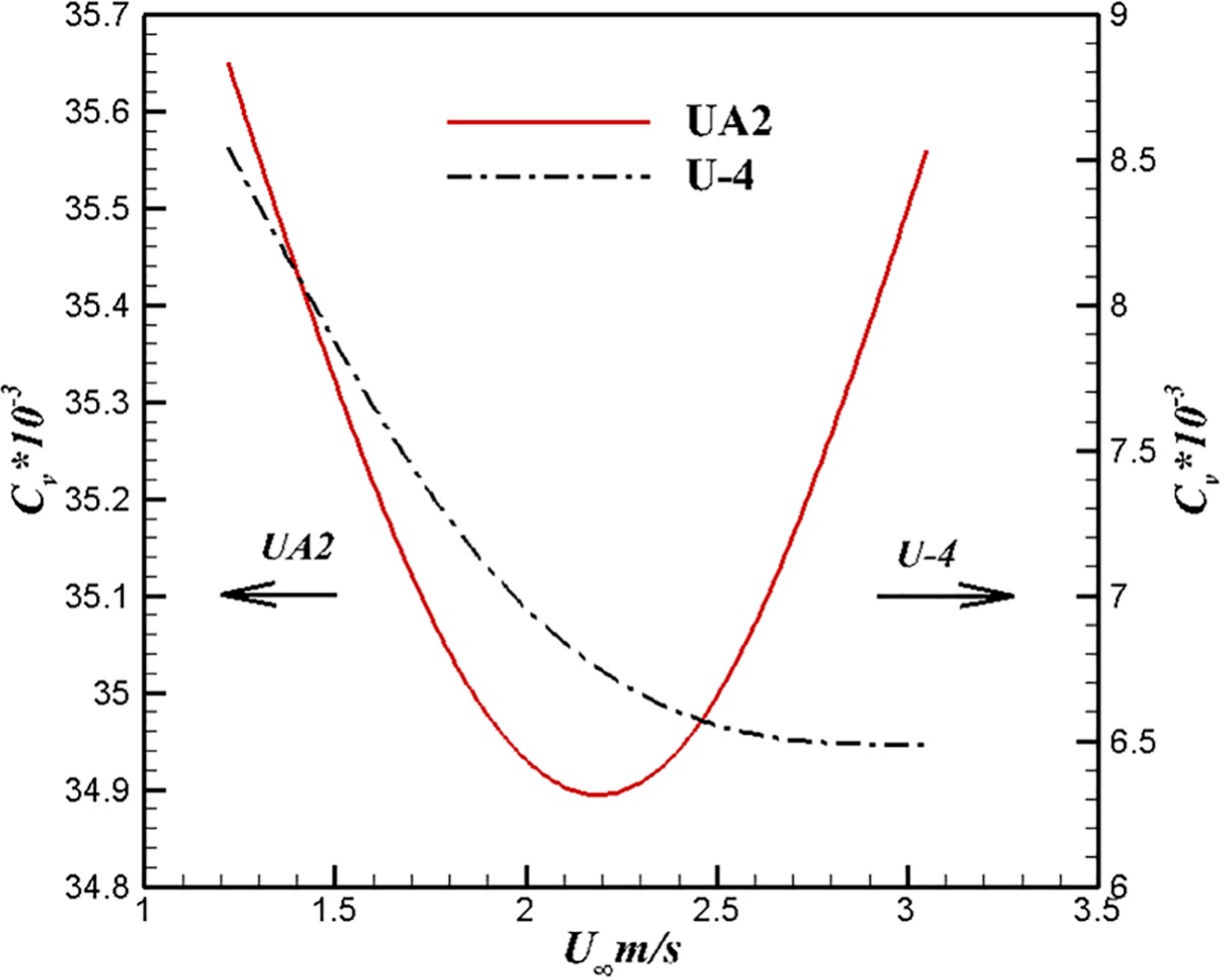
Comparison of U-4 and UA2 at different velocities.

**Fig 14:**
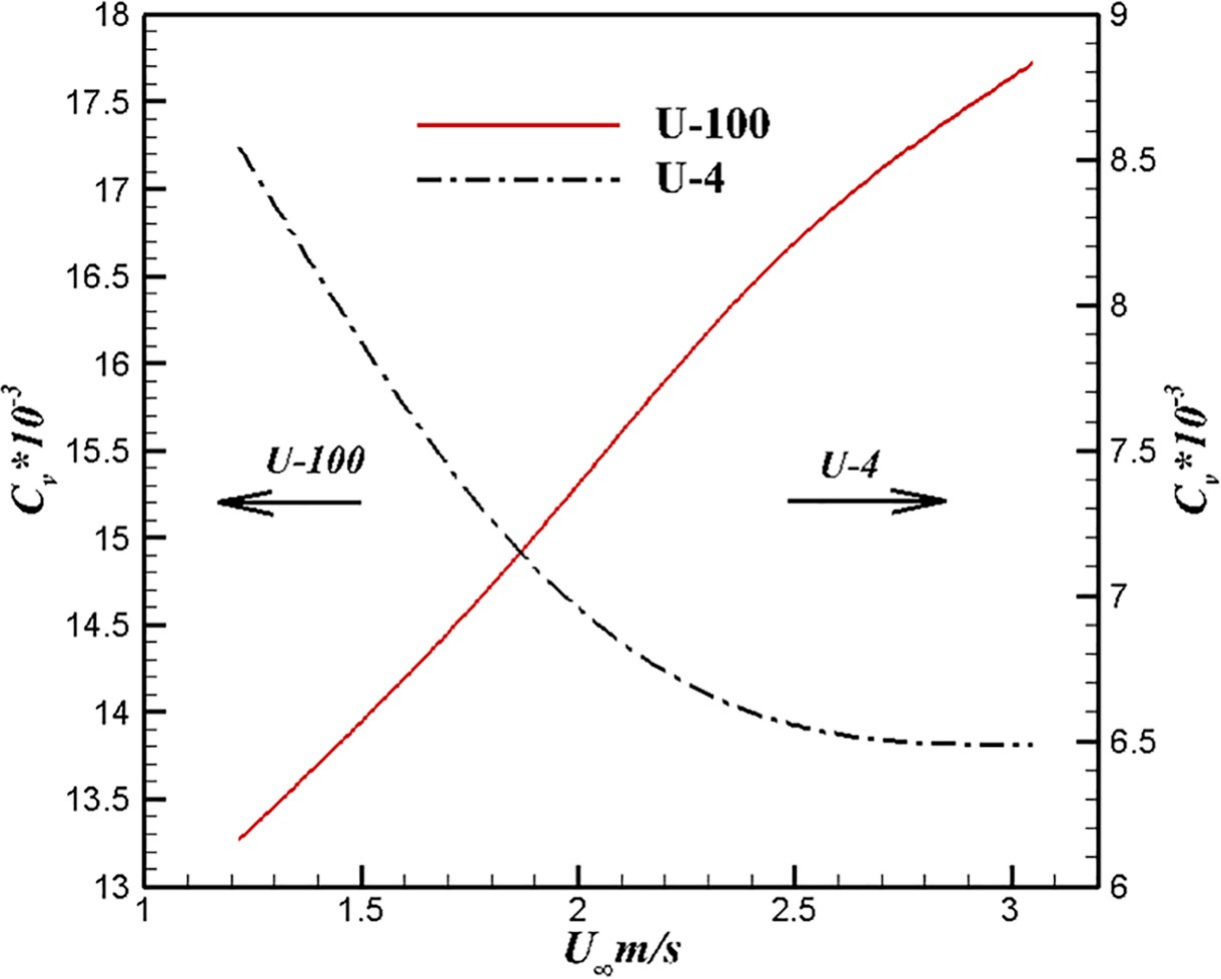
Comparison of U-4 and U-100 at different velocities.

Figs 13 and 14 show the comparison results of U-4, U-100 and UA2 at different velocities. The trend of drag coefficient is that reduction of volumetric drag coefficient with velocity increases for the body of U-4. The volume drag coefficient of UA2 decreases first and then rises. It can be seen from Fig 14 that the volume drag coefficient of U-100 increases with the increase of velocity within this velocity range. Both laminar drag coefficient and turbulent drag coefficient decrease with the increase of Reynolds number. Turbulence drag is much greater than laminar drag under the same conditions. The drag coefficient may increase with drag which is caused by laminar flow to turbulence sufficiently to destroy this variety characteristic in separate laminar or turbulent flow.

**Fig 15:**
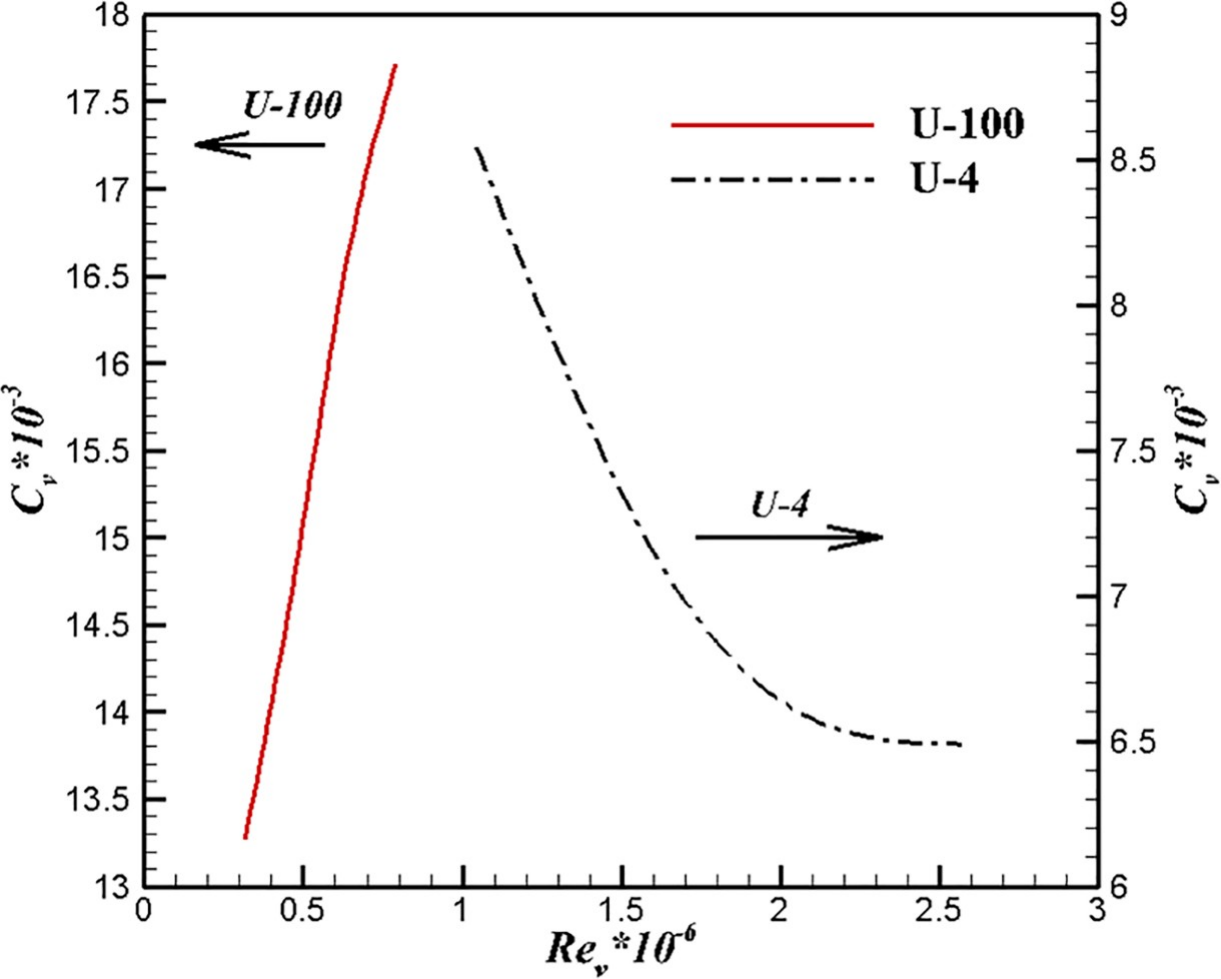
Comparison of U-4 and U-100 at different volume Reynolds numbers.

**Fig 16:**
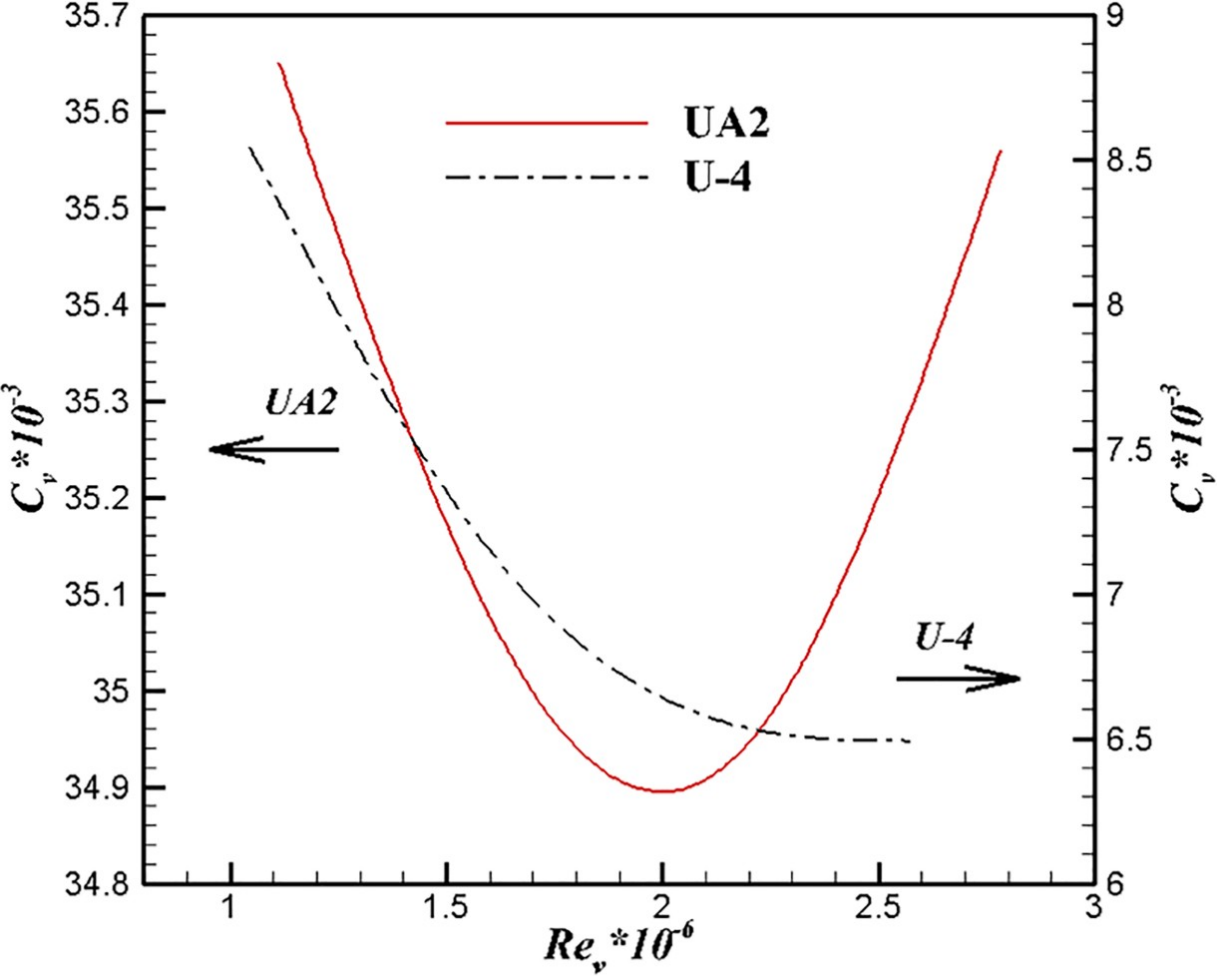
Comparison of U-4 and U-100 at different volume Reynolds numbers.

Figs 15 and 16 show the comparison results of U-4, U-100 and UA2 at different volume Reynolds numbers. The volume drag coefficient of U-4 is lower than that of the other two bodies under the same volume Reynolds number. The trend of volume drag coefficient decrease can be maintained at high volume Reynolds number for the body of U-4.

In order to study the difference between laminar flow drag and turbulent drag. A criterion is needed to judge the drag coefficient. An ideal drag of slender laminar unseparated body of revolution can be estimated by Eq (11) [30]:

CVL=2XρU∞2V23≈4.7ReV (11)

Eq (1) is consistent with Hoerner's relationship of laminar flow drag on rotating slender bodies [31]. In Ref 10, experimental data about the drag of a standard rotating body, such as an ellipsoid is collected and analyzed.

The frictional drag on the elongated ellipsoid could be estimated based on the plate concept [31], by using the surface area S and the average turbulence drag coefficient on the plate [32]. The corresponding volumetric drag coefficient can be estimated as follows

CVT=kTSV23 (12)

kT=0.0307ReL17 (13)

ReL=U∞Lν (14)

Relevant results are shown in the Table 2. The inflow velocity is converted to the volume Reynolds number of the corresponding body. For U-4 and UA2, under the same volume Reynolds number, the laminar coverage of U-4 is about twice that of UA2. Flow separation occurs at x = 0.453 when the velocity of UA2 is low. x = 0.457 for the position of the laminar separation line on UA-2 is calculated by Eq (7). Where x is the ratio of the axial distance from the separation point to the head of the body to the length of the body. U-100 does not have a high laminar coverage even at low volume Reynolds number because of its large aspect ratio.

**Table 2:** Results of different type of bodies (L is the length ratio and S is the area ratio).

Type	Re_v_×10^−6^	Ratio of Laminar Flow	*C_V_*
U-4	2.572901	68.6%(L) 72.8%(S)	0.00649
	2.092626	72.3%(L) 78.0%(S)	0.00658
	1.569470	74.2%(L) 80.5%(S)	0.00721
	1.046313	75.5%(L) 82.2%(S)	0.00854
UA2	2.782717	31.7%(L) 20.2%(S)	0.03556
	2.226174	41.3%(L) 34.4%(S)	0.03496
	1.669630	45.3%(L) 40.7%(S)	0.03502
	1.113087	45.3%(L) 40.7%(S)	0.03565
U-100	0.791586	50.7%(L) 53.7%(S)	0.01772
	0.633269	58.3%(L) 58.7%(S)	0.01655
	0.474951	64.5%(L) 69.2%(S)	0.01481
	0.316634	66.8%(L) 73.1%(S)	0.01327

The comparison of different drag coefficients is shown in Table 3. The turbulence drag coefficient is about 7 times of laminar drag coefficient. The drag coefficient increases when laminar flow transits to turbulence. This also explains the phenomenon that the drag coefficient does not decrease but increases in the previous paper. The abnormal drag coefficient of UA2 body may be caused by the flow separation on the surface of the body. It also proves the importance of inhibiting separation. As discussed above, the first method represented by U-4 shows a better performance in drag reduction.

**Table 3:** Comparison of different drag coefficients.

Type	*U*_∞_ (m/s)	*C_V_*	*C_VL_*	*C_VT_*
U-4	3.05	0.00649	0.00293	0.01957
	2.44	0.00658	0.00325	0.02021
	1.83	0.00721	0.00375	0.02106
	1.22	0.00854	0.00459	0.02231
UA2	3.05	0.03556	0.00282	0.01971
	2.44	0.03496	0.00315	0.02035
	1.83	0.03502	0.00364	0.02120
	1.22	0.03565	0.00445	0.02247
U-100	3.05	0.01772	0.00528	0.03520
	2.44	0.01655	0.00591	0.03634
	1.83	0.01481	0.00682	0.03786
	1.22	0.01327	0.00835	0.04012

### CFD results for the U-13

**Fig 17:**
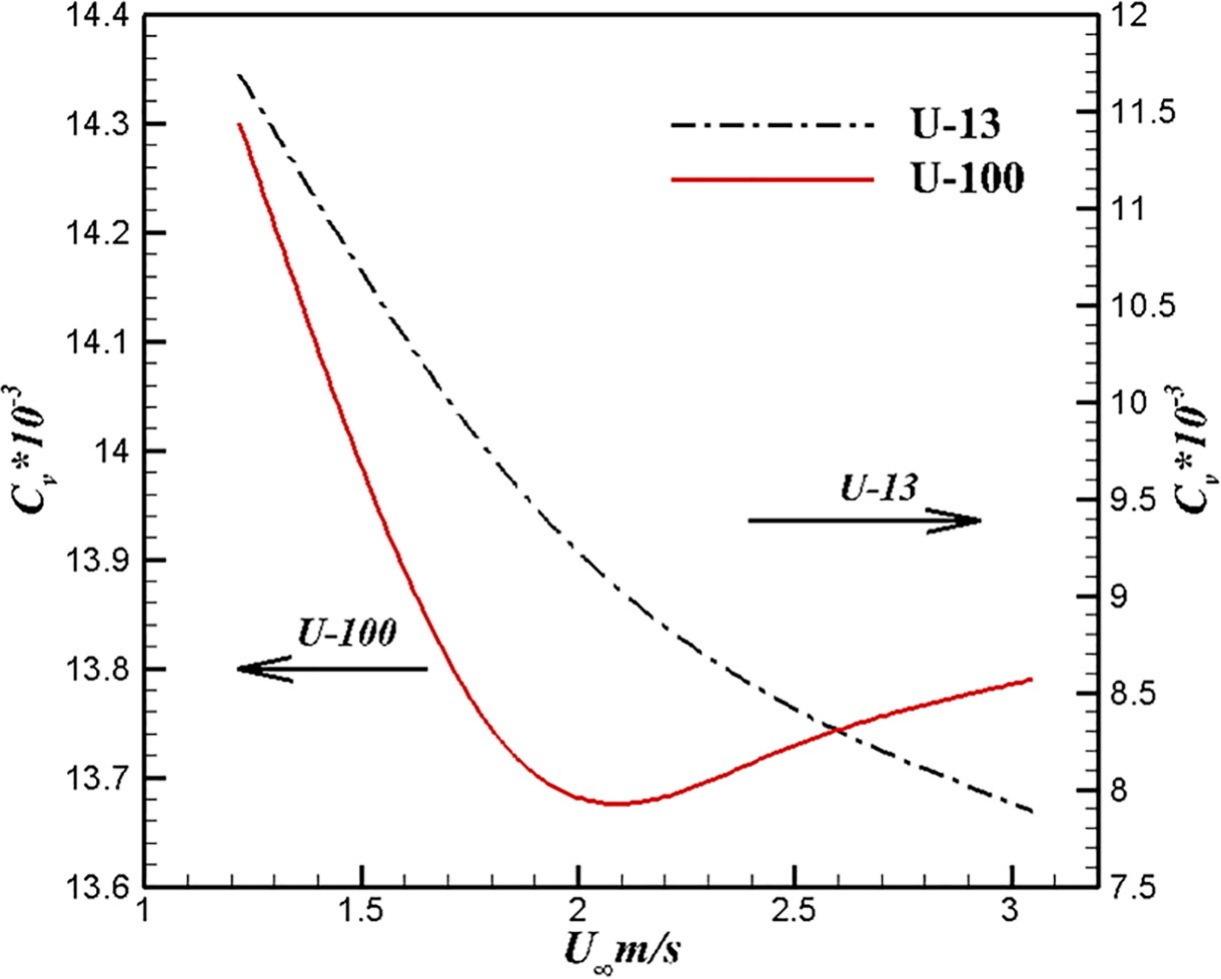
Comparison of volumetric drag coefficients at different velocities.

In this section, we discuss the performance of U-13 and U-100 in water at zero attack of angle. We perform the calculation at the ambient flow velocities 3.05m/s, 2.44m/s, 1.83m/s and 1.22m/s respectively. Computational conditions for comparing body are listed in the Table 4. The comparison of volumetric drag coefficients at different velocities are shown in Fig 17. Within the computational velocity range, the volumetric drag coefficients of U-100 first decreases and then increases with the increase of velocity and the volume drag coefficient of U-13 decreases with the increase of velocity. Increased turbulent coverage area which because of the growth of velocity results in a rise in the volumetric drag coefficient. We attribute this to the result of a surface that covers a certain proportion of turbulence. Under the same velocity, the volume drag coefficient of U-13 is less than that of U-100. Although the U-100 uses a larger aspect ratio to suppress separation and reduce surface drag, U-13 still has better performance than U-100 at the same speed. The advantage of the first method is obvious, separation can be effectively inhibited under the small aspect ratio condition by adjusting the position of maximum diameter point. Flexible design rules enable the first method to design the body with better performance under certain working conditions.

**Table 4:** Computational conditions for comparing body.

Type	Length (m)	Maximum Diameter (m)	Water depths (m)
U-13	1.5	0.122m	2.5m
U-100	1.5	0.067m	2.5m

**Fig 18:**
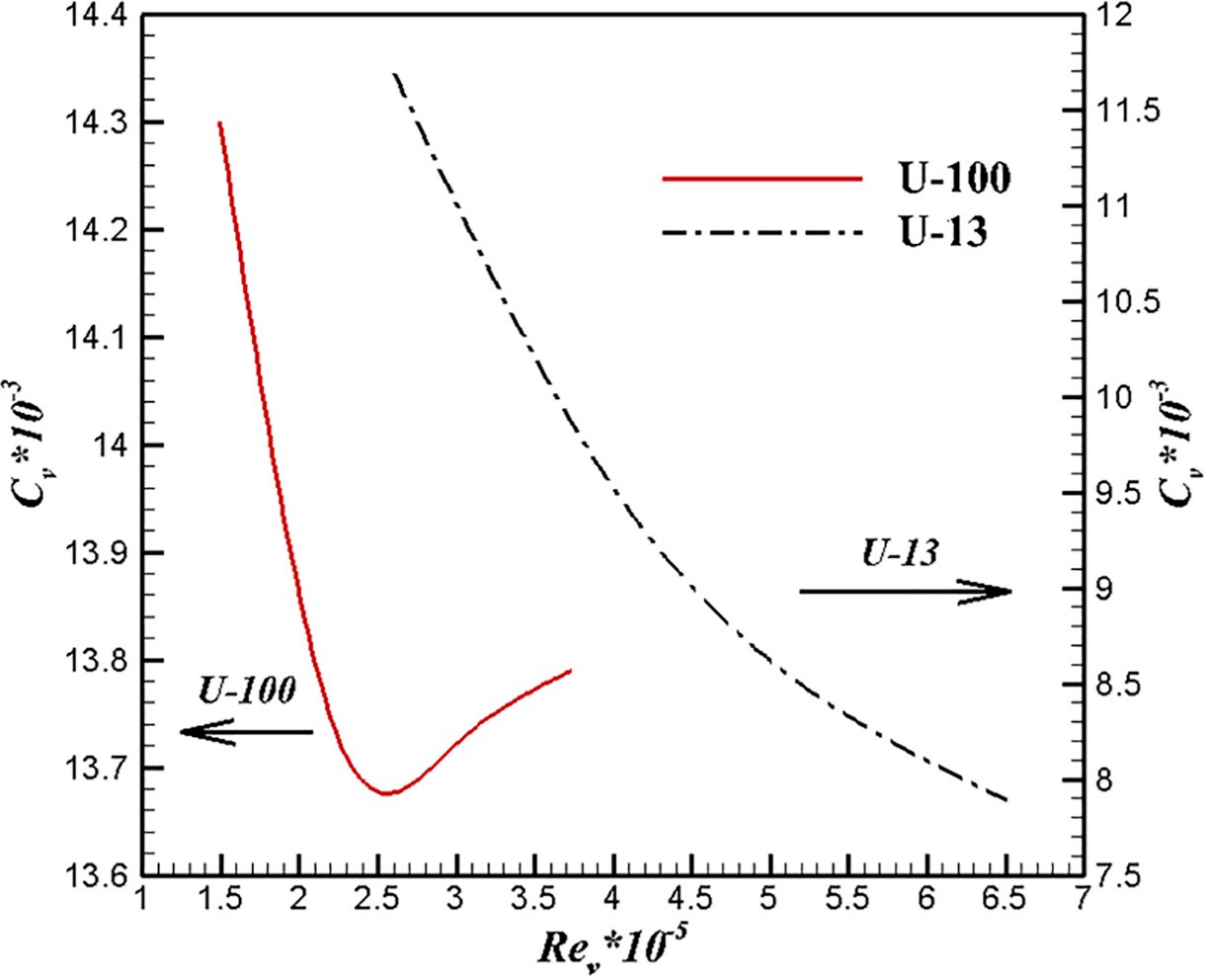
Comparisons of volumetric drag coefficients under different volume Reynolds numbers.

Fig 18 shows the results of volumetric drag coefficients under different volume Reynolds numbers. For the same type of body, the volume Reynolds number varies linearly with velocity. Obviously, the variation of volume drag coefficient with volume Reynolds number is consistent with that with velocity for the same type of body. Fig 18 shows U-13 is able to make volume drag coefficient under a lower level even under higher volume Reynolds number compared with U-100. This means U-13 can maintain a high laminar coverage even at a higher volume Reynolds number from the previous analysis. At the same time, it is obvious that the body type designed by the first method can achieve higher performance under the condition of higher volume Reynolds number.

**Fig 19:**
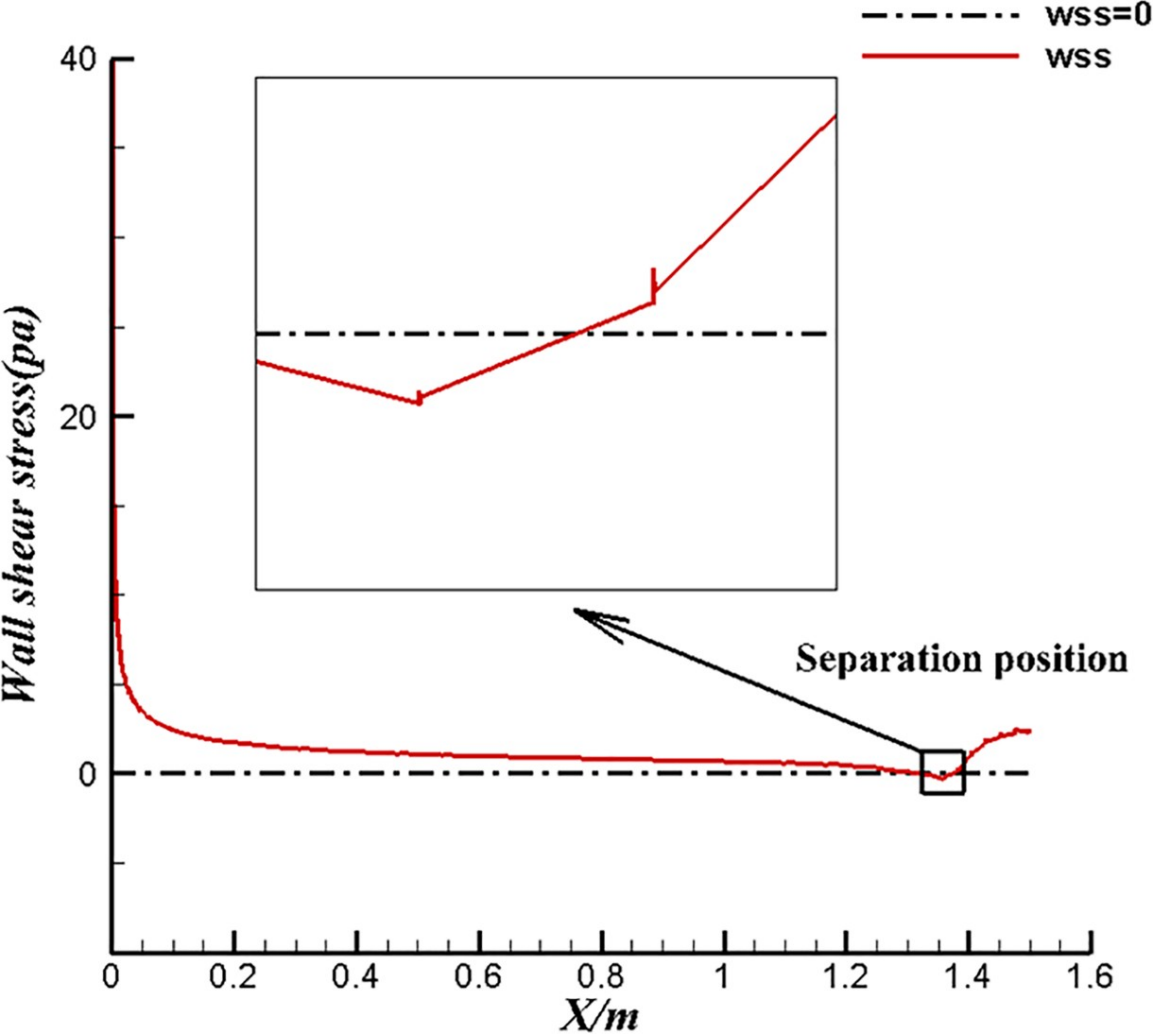
Wall Shear Stress of U-13 at 1.22m/s.

**Fig 20:**
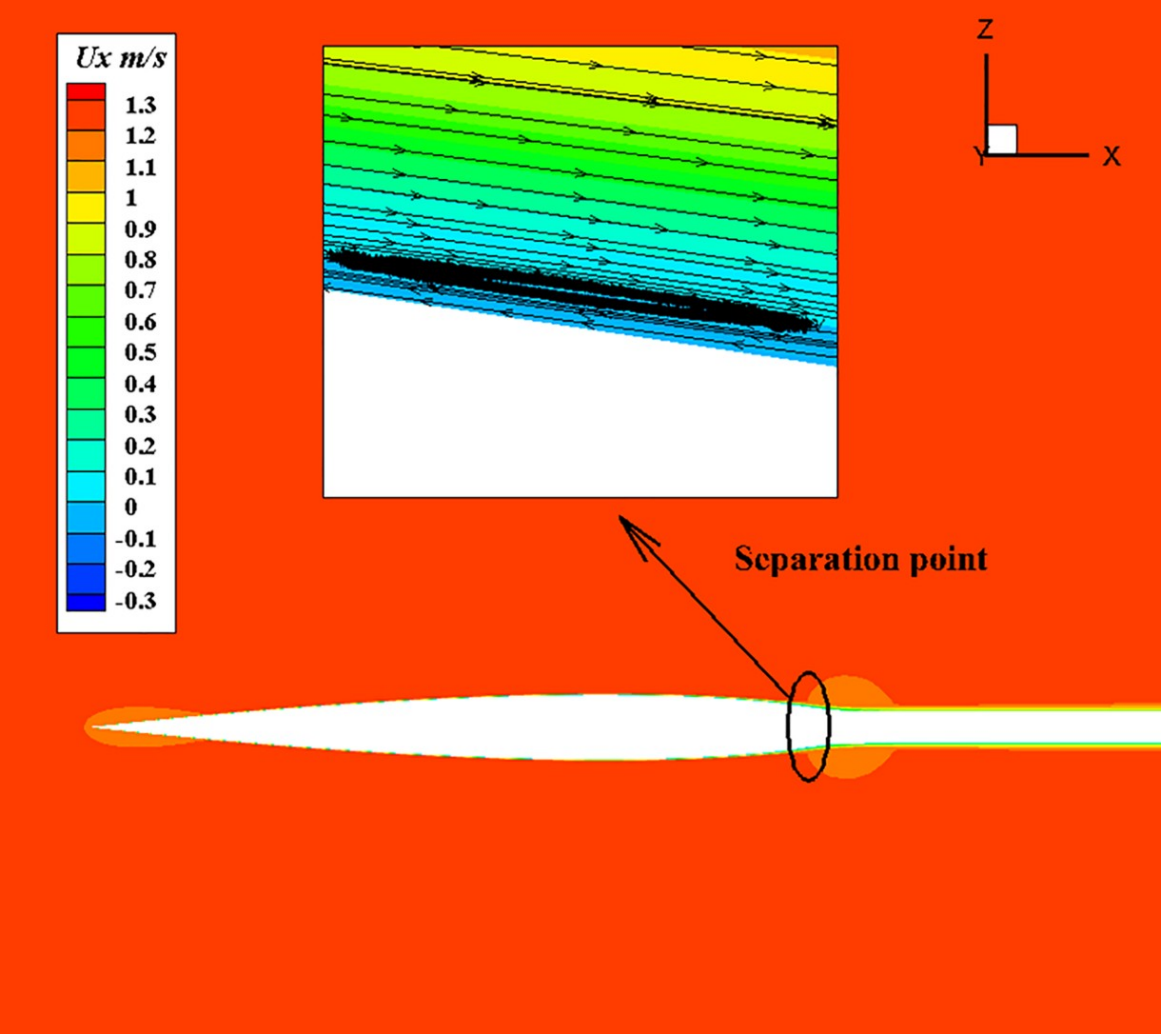
Contours of the axial velocity of U-13 at 1.22m/s.

**Fig 21:**
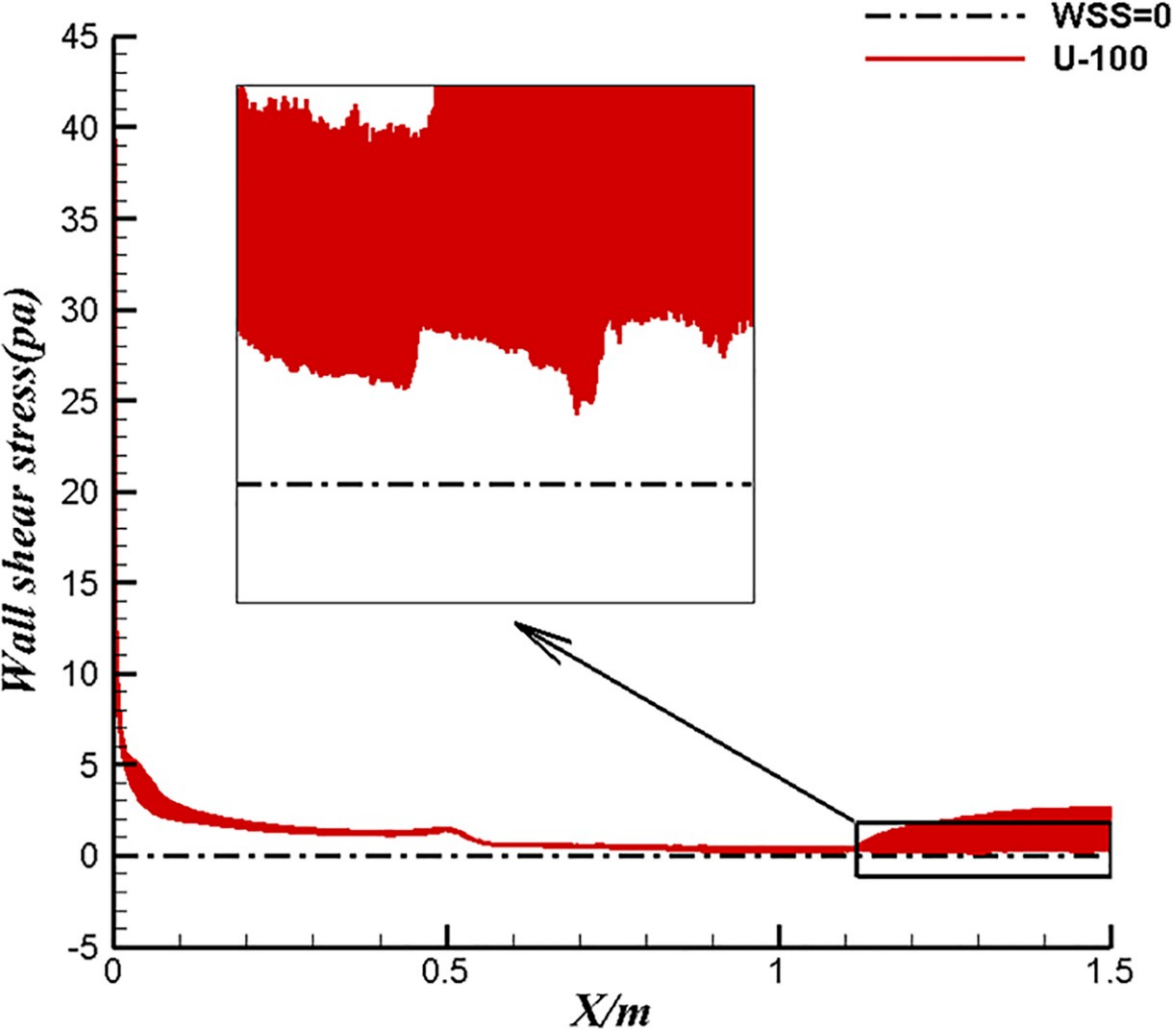
Wall Shear Stress of U-100 at 1.22m/s.

The surface flow results of different types of body are listed in the Table 5. The inflow velocity is converted to the volume Reynolds number of the corresponding body according to Eq (10). Firstly, the volume drag coefficient of U-13 is lower than that of U-100 under the same volume Reynolds number condition. As previously studied, U-13 has higher laminar flow coverage and better drag reduction under the same volume Reynolds number. For the case of 1.22 m/s, the reason why the drag coefficient of U-13 is high and the coverage of surface laminar flow is close to that of U-100 is that, the laminar separation phenomenon occurs in the tail section of U-13 at the case of 1.22m/s and such deduction could be prove by Figs 19 and 20. Separation is not detected in U-100 because of large aspect ratio as shown in Fig 21. However, even if separation occurs, the drag coefficient of U-13 is 30% lower than that of U-100. It is can be seen from Table 5 that the volumetric drag coefficient decreases as the volume Reynolds number or velocity increases when laminar flow coverage is high. the volume drag coefficient will gradually restore to the order of turbulent drag coefficient as the velocity increases when laminar flow coverage falls below a certain value between 82.1% and 89.8%. Both laminar drag coefficient and turbulent drag coefficient decrease with the increase of Reynolds number. Turbulence drag coefficient is much higher than laminar drag coefficient. The drag coefficient will change from the magnitude of laminar flow to the magnitude of turbulence when the turbulence increases. The success of U-13 also proves that the body designed by the first method is able to maintain low drag coefficient and high efficiency at high velocity.

**Table 5:** Results of different type of bodies.

Type	Re_v_×10^−5^	Ratio of Laminar Flow	*C_v_*
U-13	6.51005	87.7%(L) 89.9%(S)	0.00789
	5.20804	89.0%(L) 91.3%(S)	0.00849
	3.90603	90.6%(L) 92.8%(S)	0.00964
	2.60402	93.5%(L) 95.3%(S)	0.01169
U-100	3.73298	69.3%(L) 79.2%(S)	0.01379
	2.98638	72.7%(L) 82.1%(S)	0.01372
	2.23979	83.3%(L) 89.8%(S)	0.01373
	1.49319	88.6%(L) 93.1%(S)	0.01430

## Conclusion

In this paper, CFD is used to calculate and simulate the body design of two negative pressure gradient design methods. The steady-state model of the Transition SST model is used to calculate the pressure distribution, wall shear stress, and drag coefficient under zero angle of attack at different velocities. We evaluated four bodies designed by two methods, U-4, U-13, U-100 and UA2. Our results show that the first method is superior to Hansen in drag reduction and the drag reduction of missile type is more than 10% for the same design standard. The types of body designed by the first method is more likely to obtain the characteristics of suppressing or eliminating separation to prevent the sudden rise of drag and can effectively improve laminar flow coverage to achieve drag reduction and keep the variation characteristics of laminar flow drag coefficient under higher Reynolds number conditions. The first method is more flexible than the second method in suppressing separation and producing better drag reduction effect. The discriminant formula of separation point can effectively discriminate the location of separation point. The influence of separation can be effectively avoided in the design process by discriminant formula. The first method would be a successful method for optimum design of missile shape. This successful design method is expected to open up a promising prospect for its application in the design of small drag, noise subsonic hydrodynamic hull and underwater vehicle.

## Supporting information

S1 Table Appendix. SI UNITS AND NOMENCLATURE.
(DOCX)
