# Supplementary material for: Comparison of design methods for negative pressure gradient rotary bodies: A CFD study
Source: PLoS One. 2020 Jan 30;15(1):e0228186. doi: 10.1371/journal.pone.0228186 (PMC6992229; doi:10.1371/journal.pone.0228186)
Supplement: S1 Table — SI UNITS AND NOMENCLATURE. (DOCX) [file pone.0228186.s001.docx]

# S1 Table

# Appendix. SI UNITS AND NOMENCLATURE

| Quantity | Symbol | Units |
| --- | --- | --- |
| Length | L | m |
| Density | ρ | kg/m^3^ |
| Time | t | s |
| Velocity | v | m/s |
| Pressure | p | N/m^2^(pa) |
| Dynamic viscosity | μ | kg/(m·s) |
| Force | F | kg·m/s^2^(N) |
| Wall shear stress | τ | N/m^2^(pa) |
